# Effect of Schwertmannite Surface Modification by Surfactants on Adhesion of Acidophilic Bacteria

**DOI:** 10.3390/microorganisms8111725

**Published:** 2020-11-04

**Authors:** Agnieszka Pawlowska, Zygmunt Sadowski

**Affiliations:** Department of Chemical Engineering, Wroclaw University of Science and Technology, Wybrzeze Wyspianskiego 27, 50-370 Wroclaw, Poland; zygmunt.sadowski@pwr.edu.pl

**Keywords:** schwertmannite, acidophilic bacteria, adhesion, surface energy, surfactant, rhamnolipid, DLVO theory

## Abstract

Bacterial cell adhesion onto mineral surfaces is important in a broad spectrum of processes, including bioweathering, bioleaching, and bacterial cell transport in the soil. Despite many research efforts, a detailed explanation is still lacking. This work investigates the role of surface-active compounds, cetyltrimethylammonium bromide (CTAB), sodium dodecyl sulfate (SDS), and pure rhamnolipid (RH), in the process of bacteria attachment on the schwertmannite surface. The surface energy was calculated based on the wettability of the tested systems, and for bacteria it was 54.8 mJ/m^2^, schwertmannite-SDS 54.4 mJ/m^2^, schwertmannite-CTAB 55.4 mJ/m^2^, and schwertmannite-RH 39.7 mJ/m^2^. The total energy of adhesion estimated based on thermodynamic data was found to be negative, suggesting favorable conditions for adhesion for all examined suspensions. However, including electrostatic interactions allowed for a more precise description of bacterial adhesion under the tested conditions. The theoretical analysis using the extended Derjaguin-Landau-Verwey-Overbeek (DLVO) approach showed a negative value of total adsorption energy only in bacteria-mineral suspensions, where SDS and rhamnolipid were added. The calculated data were in good agreement with experimental results indicating the significance of electrostatic forces in adsorption.

## 1. Introduction

**Figure 1:**
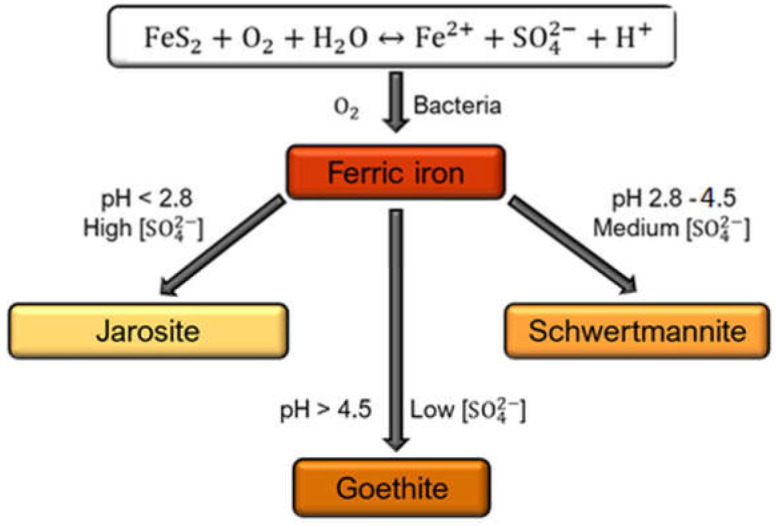
Mineral phase formation depending on acidity and sulfate concentration.

Schwertmannite, a finely crystalline ferric oxyhydroxysulfate mineral, is one of the products of the pyrite and arsenopyrite bioleaching process. The chemical composition is as follows Fe_8_O_8_(OH)_8−2x_(SO_4_)_x_, where 1 ≤ x ≤ 1.75 [1]. The share of individual mineral phases in the sludge generated by post-mining water reservoirs depends on the acidity of water and sulfate ion concentration (Figure 1). It has been established that the schwertmannite particles play an essential role in controlling the transport and adsorption of various contaminants in acid mine drainage (AMD) [2] and, together with ferrihydrite, schwertmannite is one of the most frequently observed minerals found in old-mine acidic waters. Biosynthesis of schwertmannite by *Acidithiobacillus ferrooxidans* occurs in Fe-reach sulfate solution in the pH range of 2.8–4.5 [3]. The chemical synthesis of schwertmannite is strictly dependent on the pH and the Fe(II)/Fe(III) ratio. The preferred pH range is 2.5–3.5, and the molar ratio equals 0.5 [4]. There are significant differences in the structure between chemically and biologically synthesized schwertmannite. Chemically synthesized schwertmannite can spontaneously transform to goethite or jarosite. Biologically synthesized schwertmannite is metastable compared to goethite and has a much larger specific surface area (58.79 m^2^/g) than the mineral synthesized chemically (6.31 m^2^/g) [5]. Wang and colleagues [2] investigated the transformation of schwertmannite to jarosite in the presence of NH^4+^ ions and iron oxidation bacteria.

The schwertmannite surface can often be modified by the adsorption of various ions and both inorganic and organic substances. Polysaccharides and biosurfactants produced by bacteria adsorbed on the mineral surface can facilitate cell adhesion and increase the heterocoagulation of bacteria and small mineral particles.

The chemical synthesis or biosynthesis of schwertmannite requires a strict pH and temperature because surface changes might occur. An increase in temperature up to 100 °C causes goethite precipitation. Miyata and co-workers [1] obtained 90% pure goethite using the acidophilic Fe(II)-oxidizing betaproteobacterium strain GJ-E10, at 37 °C, with a pH equal to 3.5. The Fe^2+^ oxidation rate affected secondary iron hydroxy sulfate mineral precipitation [6]. The slow bio-oxidation treatment led to jarosite precipitation. On the contrary, rapid oxidation treatment using H_2_O_2_ resulted in the precipitation of schwertmannite.

Processes such as bioleaching, biocorrosion, and bioprecipitation are closely related to microbial cell attachment to the mineral surface [7]. Bacterial adhesion can be explained by surface thermodynamics and the Derjaguin-Landau-Verwey-Overbeek (DLVO) theory. The thermodynamic interpretation assumes that the spontaneous attachment of the microbial cell to the mineral surface occurs when the free energy per unit area (ΔG_adh_) is negative. According to the Dupre equation:

ΔG_BSL_ = γ_BS_ − γ_BL_ − γ_SL_ (1)

This means that if the interfacial energy (γ_BS_) of the bacteria (B) and the mineral surface (S) is smaller than a sum of bacteria and liquid (γ_BL_) and the mineral surface and liquid interface (γ_SL_), the adhesion of the bacterial cell will be favored [8,9]. In other words, the degree of bacterial cell adhesion will increase as the difference in surface energy between the bacterial cell and the mineral surface decreases [10]. According to the van Oss-Chaudhury-Good equation, the surface free energy of solid is related to the liquid-solid contact angle [11,12].

(2) $$\left(1-\cos\Theta \right)\gamma_{L}=2{\left(\gamma_{S}^{\mathrm{LW}}\gamma_{L}^{\mathrm{LW}}\right)}^{1/2}+{\left(\gamma_{S}^{+}\gamma_{L}^{-}\right)}^{1/2}+{\left(\gamma_{S}^{-}\gamma_{L}^{+}\right)}^{1/2}$$

where Θ is the contact angle of solid, γ^LW^ is the Lifshitz-van der Waals component of the surface energy, and γ^+^ and γ^−^ are the electron-acceptor and electron-donor components of free energy (S-solid; L-liquid).

For calculation of the total free energy of bacterial cell adhesion, it is necessary to know the interface energy of bacteria-mineral (γ_BS_), bacteria-liquid (γ_BL_), and mineral-liquid (γ_SL_). The free interface energy consists of a component related to Lifshitz-van der Waals (γ^LW^) and acid-base interactions (γ^AB^). These components can be calculated using the following equations:

(3) $$\gamma_{\mathrm{BS}}^{\mathrm{LW}}=\gamma_{B}^{\mathrm{LW}}+{\;\gamma }_{S}^{\mathrm{LW}}\;-\;2\sqrt{\gamma_{B}^{\mathrm{LW}}\gamma_{S}^{\mathrm{LW}}}$$

(4) $$\gamma_{\mathrm{BS}}^{\mathrm{AB}}\;=\;2\;(\sqrt{\gamma_{B}^{+}\gamma_{B}^{-}}\;+\;\sqrt{\gamma_{S}^{+}\gamma_{S}^{-}}\;-\;\sqrt{\gamma_{B}^{+}\gamma_{S}^{-}}\;-\;\sqrt{\gamma_{B}^{-}\gamma_{S}^{+}})$$

The thermodynamic surface theory does not consider the electrostatic interaction between bacterial cells and the mineral surface. The DLVO theory incorporates these interactions into a total adhesion energy calculation. The main success of the DLVO theory created by Derjaguin and Landau and Verwey and Overbeek was the description of colloid stability. Assuming that the bacterial cell and mineral particles have a colloidal size, the DLVO theory can be used to describe the interaction between these two objects. According to the DLVO theory, the total interaction energy is a sum of electrostatic interaction energy (G^EL^) and Lifshitz-van der Waals attractive energy (G^LW^) [7]. Because the bacterial cell should be considered biocolloid, additional energy of interaction must be considered, resulting from the acid-base hydrophobic interactions and steric interaction (extended DLVO theory). Thus, the total energy interaction of the bacterial cell-mineral particle is equaled:

G^TOT^ = G^EL^ + G^LW^ + G^AB^ (5)

where G^EL^ is the electrostatic interaction energy, G^AB^ is the acid-base interaction energy, and GLW is the Lifshitz-van der Waals attractive energy. When G^TOT^ is negative, there is an attraction between the bacterial cell and the mineral surface and cell adhesion takes place. Equation (6) allows to determine electrostatic interaction energy GEL between the spherical bacterial cell and the flat mineral surface immersed in water:

(6) $$G^{\mathrm{EL}}\left(H\right)={\pi \varepsilon }_{0}\varepsilon a\left(\zeta_{B}^{2}+\zeta_{S}^{2}\right)\left\{\frac{2\zeta_{B}+\zeta_{S}}{\zeta_{B}^{2}+\zeta_{S}^{2}}\ln\frac{1+\exp\left(-\kappa H\right)}{1-\exp\left(-\kappa H\right)}+\ln\left[1-\exp\left(-\kappa H\right)\right]\right\}$$

where ε_0_ is the dielectric permittivity of free space, ε is the relative dielectric permittivity of the medium (80 for water), R is the bacteria radius, and 1/κ is the Debye-Hückel length. $\zeta_{B}\;$ and $\zeta_{S}\;$ refer to the zeta potentials of the bacterium and the mineral surface, respectively [13].

The Lifshitz-van der Waals component of total interaction energy can be calculated using the following equation:

(7) $$G^{\mathrm{LW}}\;=\;-\frac{\mathrm{AR}}{6H^{2}}$$

where A is the Hamaker constant for the bacteria (B), water (L) and mineral (S) (calculated from Equation (8)) and H is the distance.

(8) $$A_{\mathrm{BSL}}\;=\;24\;\pi x_{0}^{2}\left(\sqrt{\gamma_{B}^{\mathrm{LW}}}\;-\;\sqrt{\gamma_{S}^{\mathrm{LW}}}\right)\;(\sqrt{\gamma_{L}^{\mathrm{LW}}}\;-\;\sqrt{\gamma_{S}^{\mathrm{LW}}})$$

where $\gamma_{\mathrm{BSL}}^{\mathrm{LW}}$ is the Lifshitz-van der Waals apolar component of bacteria surface energy (B), mineral (S), and water (L), respectively.

(9) $$G^{\mathrm{AB}}\;=\;2{\pi R\lambda G}_{\mathrm{BSL}\;}^{\mathrm{AB}}e^{\frac{H_{0}\;-\;H}{\lambda }}$$

where λ is the decay length of water molecules, approximately 0.2 nm for pure water, and H_0_ is equal to 0.157 nm [14].

(10) $$\Delta G_{\mathrm{BSW}}^{\mathrm{AB}}=2[\sqrt{\gamma_{L}^{+}}(\sqrt{\gamma_{B}^{-}}+\sqrt{\gamma_{S}^{-}}-\sqrt{\gamma_{L}^{-}})+\sqrt{\gamma_{L}^{-}}(\sqrt{\gamma_{B}^{+}}+\sqrt{\gamma_{S}^{+}}-\sqrt{\gamma_{L}^{-}})-\sqrt{\gamma_{B}^{+}\cdot \gamma_{S}^{-}}-\sqrt{\gamma_{B}^{-}\cdot \gamma_{S}^{+}}]$$

where $\gamma_{B,S,L}^{+}$ and $\gamma_{B,S,L}^{\_}$ are donor and acceptor components of acid-base interactions.

Cell-surface interaction forces can be determined using atomic force microscopy (AFM). The adhesion forces between sulfide minerals and acidophilic bacteria have been measured previously [8,15,16,17]. The adhesion force between the AFM tip and the bacteria surface was in the range of 3.9 to 4.3 nN [18].

Studies of *Bacillus subtilis* adhesion onto the gibbsite (γ-AlOOH) surface using ATR-FTIR spectroscopy indicate that chemical interactions also participate in the adhesion. It was found that changes in protein conformation led to the formation of a chemical bond between bacterial surface groups and hydroxyl on the gibbsite surface [19].

Modification of mineral surface through adsorption of biosurfactants and biopolymers is an essential factor in the process of cell adhesion. Polymers produced by microorganisms can inhibit or promote adhesion, depending on their affinity to the mineral surface. It was presented that extracellular polymer substances (EPSs) are responsible for attaching *Acidithiobacillus ferrooxidans* cells to the chalcopyrite surface [20] and pyrite [21] and, depending on culture conditions, show different strengths of adhesion, which suggests that the composition of extracellular polymers influences the adhesion process. Schwertmannite was chosen as a mineral, whose presence increases bacterial colonization in an acidic environment, accelerating the bioleaching process.

There have been few articles studying the effects of surfactants on both minerals and bacteria, incorporating the evaluation using different physical-chemical approaches, explaining processes occurring in the natural environment. Therefore, the article aims to explain the role of surface-active compounds in the adhesion of bacteria to the schwertmannite surface in acidic conditions, naturally occurring in acid main drainage. The adsorption of bio- and surfactants causes changes of the surface free energy, indicating conditions favorable for adhesion. The free energy of bacterial adhesion to the modified mineral surface was calculated. The extended DLVO theory was applied to determine the interaction energy between the bacterial cell and schwertmannite surface. Information about the initial step of a bacteria-mineral interaction in the presence of surface-active compounds is critical for developing innovative solutions in mineral processing.

## 2. Materials and Methods

### 2.1. Bacteria Isolation and Growth

Chemoautotrophic acidophilic bacteria were isolated from acidic waters formed in excavations of former pyrite mines (Poland). The water sample was introduced to a 100-mL liquid medium and incubated at 35 °C (150 rpm) for up to 3 weeks. 16S rRNA gene sequence analysis revealed the dominance of *Acidithiobacillus ferooxidans* (95.37%) and the presence of 4.63% of unclassified species. For isolation and bacterial growth, 9K medium with the following composition was used (grams per liter of deionized water): 44.8 g FeSO_4_·7H_2_O, 3.0 g (NH_4_)_2_SO_4_, 0.5 g K_2_HPO_4_, 0.5 g MgSO_4_·7H_2_O, 0.1 g KCl, 0.01 g Ca(NO_3_)_2_. The medium was maintained at pH 2 with 5 M H_2_SO_4_. The iron sulfate solution was sterilized by filtration through a 0.45-μm membrane filter, while the other components in the autoclave were heated at 125 °C for 15 min. Bacterial cultures for adsorption experiments were carried out in Erlenmeyer flasks filled with 100 mL of medium and 10% *v*/*v* inoculum (35 °C, 150 rpm). Microorganisms harvested at the end of the exponential growth phase were filtered through the paper filter to remove precipitates and centrifuged (5000 rpm). The water suspension of bacterial cells was contacted with the schwertmannite at different surfactant and rhamnolipid concentrations.

### 2.2. Synthesis of Schwertmannite

Schwertmannite synthesis was carried out in an acidic environment (pH 3) using ferrous chloride tetrahydrate. Higher pH causes precipitation of iron compounds [3]. FeCl_2_·4H_2_O (17.28 g) was dissolved in 800 mL of deionized water and heated to 60–65 °C. Then, 5 mL of H_2_O_2_ and 4.8 g of Na_2_SO_4_ were added. The suspension was heated again at 60 °C for 15 min. After cooling to room temperature, the precipitate was separated. The sediment was washed several times with deionized water to reduce the high concentration of chloride ions. An FEI Quanta 250 scanning electron microscope with an EDS system was used to determine the mineral elemental analysis. The average schwertmannite sample composition was O—86.23 atomic%, S—2.40 atomic%, and Fe—11.37 atomic%.

### 2.3. Surfactants and Biosurfactant

Pure cetyltrimethylammonium bromide (CTAB, Sigma-Aldrich, St. Louis, MO, USA) was used in the experiments as a cationic surfactant and sodium dodecyl sulfate (SDS, Sigma-Aldrich, St. Louis, MO, USA) was used as an anionic surfactant. Rhamnolipid with a 90% purity was produced by AGAE Technologies, LLC (Corvallis, OR, USA).

### 2.4. Electrokinetic Measurements

The zeta potentials of schwertmannite particles and bacterial cells were determined using a Zetasizer 2000 (Malvern, UK) at constant ionic strength equal to 10^−3^ M NaCl. Suspensions were conditioned for 60 min. The zeta potential values were an average of five measurements calculated using the Smoluchowski approximation.

### 2.5. Particle Size Analysis

Particle size distribution was analyzed by laser light diffraction. The Beckman Coulter LS 13 320 analyzer (Brea, Ca, United States) equipped with a Universal Liquid Module (ULM) was used. The schwertmannite sample was introduced to the instrument and suspended in a 10^−3^ M NaCl solution. Based on the light scattering images obtained during measurement, the particle size distribution was determined.

### 2.6. Contact Angle Measurements

Contact angles of schwertmannite were measured using an imaging goniometer. The profile of a liquid drop was electronically analyzed. The bacterial cell’s contact angle was measured by placing a liquid drop on the bacterial lawn, formed by vacuum filtration of a 50 mL microorganism suspension through the membrane filter. Contact angle measurements were carried out with three liquids (water, diiodomethane, and formamide) at room temperature.

### 2.7. Stability of Schwertmannite Suspensions

The stability of the schwertmannite suspensions was investigated in the presence of surfactants and bacterial cells using a Turbiscan LabEXPERT instrument (Formulation L’Union, Toulouse, France). These apparatuses detect and measure transmission and backscattering during the suspension sedimentation. The Turbiscan Stability Index (TSI) parameter was used to characterize the stability of a mineral suspension.

## 3. Results

### 3.1. Zeta Potentials

**Figure 2:**
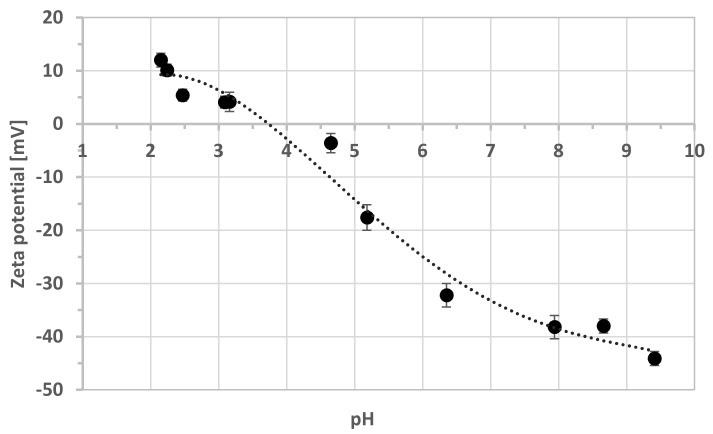
The zeta potential of schwertmannite as a function of pH (ionic strength 10^−3^ M NaCl).

The adhesion of bacterial cells to the mineral surface is dependent on various physicochemical properties of both bacterial cells and the mineral surface [22]. It is also a result of van der Waals forces, hydrophobicity, and electrostatic interaction. Dissimilar particles with opposite charges cause a strong adhesion. For this reason, the zeta potentials of mineral particles and bacterial cells were measured. Figure 2 shows the zeta potential changes in the pH function for schwertmannite particles obtained from pure chemical reagent. The iso-electric point was observed at pH 3.75. Above this value, the negative charge increased with an increasing pH and reached a maximum value of −44.1 mV at pH 9.41.

The measurements of bacteria zeta potentials carried out under the conditions that occur in AMD are shown in Table 1 and were almost constant. Earlier studies [20] have reported a similar surface charge value for acidophilic bacteria, such as *A. ferrooxidans* grown on a medium containing FeSO_4_·7H_2_O (+4.22 ± 0.52 mV, pH 2). The positive surface potential at the tested pH is due to the protonation of ammonium groups, resulting from functional groups (proteins) on the cell surface [23].

**Figure 3:**
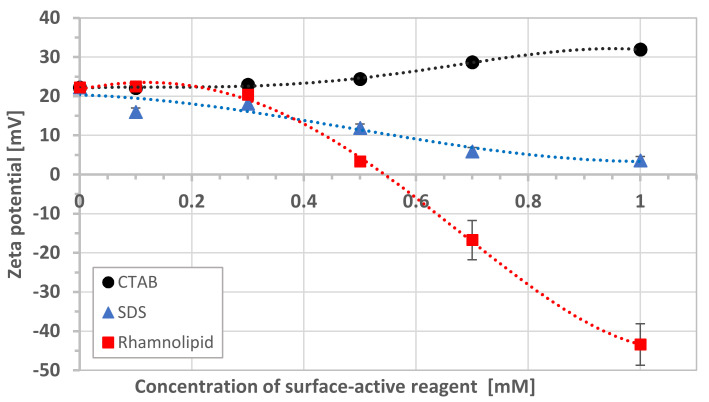
The zeta potential of schwertmannite in the presence of cetyltrimethylammonium bromide (CTAB), sodium dodecyl sulfate (SDS), and rhamnolipid (ionic strength 10^−3^ M NaCl, pH 3).

Modification of the mineral surface by surfactant and biosurfactant adsorption changed its zeta potential (Figure 3). In the case of CTAB, an increase in surfactant concentration had a slight effect on the potential (the difference was only +9.8 mV). When SDS surfactant and rhamnolipid were introduced into the suspension, a potential decrease was observed. The effect was more substantial for rhamnolipid, where the potential changed from +20 to −43.4 mV for pure mineral and a biosurfactant concentration of 10^−3^ M, respectively.

**Figure 4:**
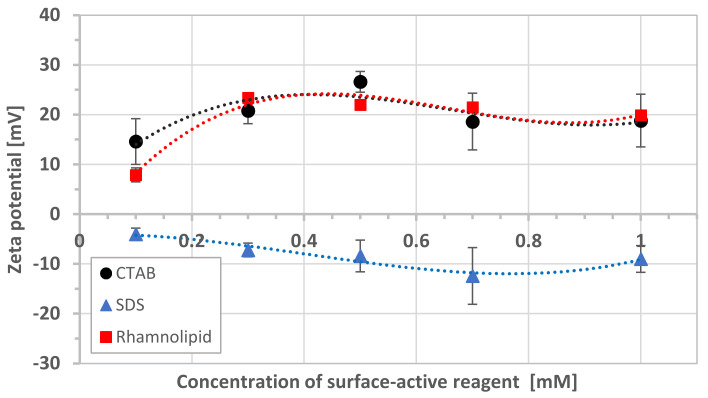
The effect of synthetic surfactants and rhamnolipid on the zeta potential of bacteria (ionic strength 10^−3^ M NaCl, pH 3).

Additionally, the effect of synthetic surfactants and rhamnolipid on the zeta potential of bacterial cells was investigated. Figure 4 shows the results of zeta potential measurements for different concentrations of surface-active reagent. The zeta potential for bacteria was about +5 mV under the experimental conditions. The addition of rhamnolipid increased the positive electric charge of the cell surface, which was consistent with the study of Lin et al. [24]. The same situation was observed for cationic surfactant. An increase in the concentration of rhamnolipid and CTAB caused only minor changes in the zeta potential. In turn, the addition of anionic surfactant caused the zeta potential of bacteria to become negative. The values increased slightly with surfactant concentration from −4.0 to −12.4, for 10^−4^ to 7 × 10^−4^ M, respectively.

### 3.2. Particle Size Distribution

The presence of surface-active substances in the aqueous suspension of schwertmannite particles might cause a change in particle size due to their aggregation. Therefore, the next step was to measure the tested systems’ particle size distribution: mineral surface-active compounds. The results are presented in Table 2. The size distribution curve of pure schwertmannite showed the following values: mean 18.20 μm, median 15.85 μm, mode 19.76 μm, and span 1.84. No significant changes in particle size were observed when CTAB and SDS were introduced into the suspension. For rhamnolipid, the largest particles were noticed for a biosurfactant concentration of 10^−3^ M (median 58.49 μm).

### 3.3. Stability Measurements

**Figure 5:**
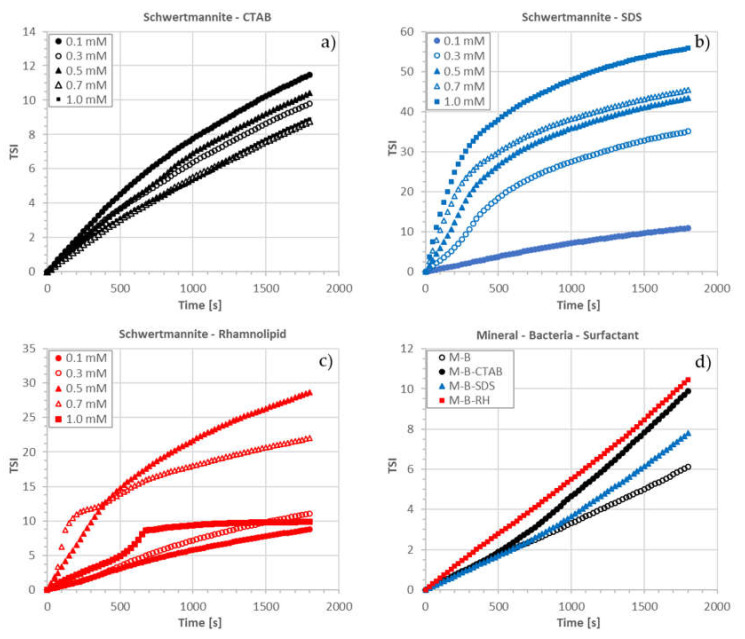
Results of stability measurements for various systems: schwertmannite (M) with (**a**) CTAB, (**b**) SDS, (**c**) rhamnolipid (RH), (**d**) bacteria and bio-/surfactant (pH 3, 10^−3^ M NaCl).

The effect of surface-active reagents on the stability of the schwertmannite suspension is presented in Figure 5a–c. The increase in the cationic surfactant (CTAB) caused a systematic increase in the stability of the schwertmannite suspension. The mineral suspension showed the highest degree of stability at 10^−3^ M of surfactant. The opposite effect was observed in the presence of anionic surfactant. With increasing concentration of SDS, the schwertmannite suspension stability decreased (Figure 5b). The schwertmannite suspension shows more interesting behavior in the presence of rhamnolipid. For the low rhamnolipid concentration (10^−4^ and 3 × 10^−4^ M), the schwertmannite suspension remains stable. At a rhamnolipid concentration of 5 × 10^−4^ and 7 × 10^−4^ M, we observed a strong destabilization effect. The high stability of the schwertmannite suspension reappeared for the rhamnolipid concentration of 10^−3^ M, which might indicate a steric stabilization effect.

Figure 5d shows how surfactants (conc. 10^−3^ M) and bacteria influence schwertmannite suspension stability. The destabilizing effect of mineral and suspension of bacteria increased in the following order: SDS, CTAB, and rhamnolipid.

### 3.4. Surface Energy

The presence of surface-active substances in the aqueous suspension of minerals caused changes in the surface free energy of schwertmannite. A single bacterial cell’s behavior on the mineral surface can be described by the thermodynamic model [11]. To apply this, it was necessary to calculate the bacteria’s free surface energy and the mineral, which were determined by measuring the contact angles (Table 3).

The total surface energy of mineral and bacteria were very similar, varying from 54.4 to 57.0 mJ/m^2^. In the case of solid modified by rhamnolipid, the components of surface energy had lower values, so the total energy was also lower compared to other systems (39.7 mJ/m^2^).

### 3.5. Bacterial Cell Adhesion—Thermodynamic Approach

Calculated free energies of bacterial cells and the schwertmannite surface (Equations (1), (3), and (4)) allowed the determination of bacterial cells’ total adhesion energy to the pure and modified mineral surface. For the estimation of $\Delta G_{\mathrm{BSL}}^{\mathrm{TOT}}$ , the Liftshitz-van der Waals $\Delta G_{\mathrm{BSL}}^{\mathrm{LW}}$ and the acid-base component $\Delta G_{\mathrm{BSL}}^{\mathrm{AB}}$ were calculated from the interfacial tensions. The results are presented in Table 4.

Calculated Liftshitz-van der Waals and acid-base components were both negative only in the case of mineral modified by rhamnolipid, resulting in a high negative value of total free energy of adhesion (−87.64 mJ/m^2^). This suggests that the maximum effective adhesion force was between the mineral, bacteria, and rhamnolipid. For the remaining components, total free energy values were in the range −14.4 to −17.22 mJ/m^2^.

### 3.6. Bacterial Cell Adhesion—DLVO Approach

The application of classical DLVO theory to the interaction between the bacterial cell and the mineral surface has not always been successful [25], mainly because the DLVO theory does not account for specific interactions, such as acid–base force, steric forces, and roughness of the mineral surface. An extended DLVO theory is replacing the traditional DLVO approach to acid–base interactions.

**Figure 6:**
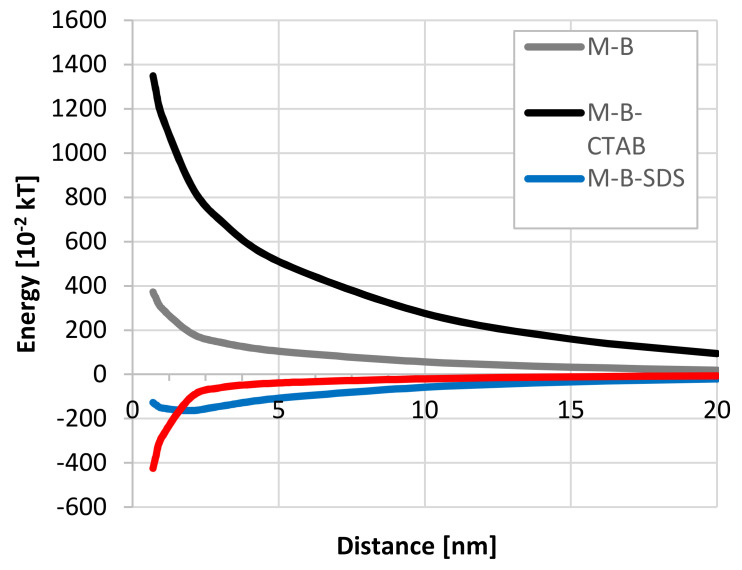
Mineral-bacteria-surfactant energy diagrams at pH 3, ionic strength 10^−3^ M.

The extended DLVO theory was used at a low ionic strength assumption (10^−3^ M). It follows that the adhesion of bacterial cells to the mineral surface can be considered as heterocoagulation. Analyzing the interaction of bacteria with schwertmannite (Figure 6), it can be concluded that there is an energy barrier, but it is not very high.

The adsorption of cationic surfactant (CTAB) caused an increase in the positive zeta potential value—both schwertmannite particles to +31.9 mV (Figure 3) and bacterial cell to +18.8 mV (Figure 4)—resulting in a high energy barrier, which inhibits the adhesion. Strong repulsion caused by the increasing cationic surfactant concentration generates higher schwertmannite suspension stability (Figure 5a). The presence of anionic surfactant only slightly reduces the positive value of zeta potential of schwertmannite from +4.1 (Figure 2) to +3.6 mV (Figure 3). The zeta potential of bacterial cells at the 10^−3^ M concentration of anionic surfactant equals a small negative value (−9.0 mV). This condition promotes heterocoagulation and adhesion (total interaction energy has negative values; Figure 6). When rhamnolipid at a concentration of 10^−3^ M was added to the schwertmannite suspension, the zeta potential’s negative value sharply increased to −43.4 mV. Under the same conditions, the zeta potential of bacterial cells reached a value of +19.9 mV—such conditions are favorable to adhesion and, therefore, to the destabilization of bacterial-mineral suspension (Figure 5d). The total energy of interaction between the bacterial cell and the schwertmannite particle has a high negative value, especially at a short distance (Figure 6).

## 4. Discussion

The biological model of bacterial cell adhesion to the solid was modified many times. An important role is played by biofilm formed on the solid surface [26]. The concept of cell adhesion by biofilm formation is correct, although it omits the cell-solid interaction. However, if microbial cell attachment to the mineral samples is considered as the colloid system, the interaction forces based on the DLVO theory will be applicable. These interactions are of different ranges and strengths, but their combined action leads to cell adhesion. The transport of bacterial cells to the mineral surface is due to a long-range interaction (50-nm distance). The main interactions that determine cell attachment to the mineral surface are the result of short-range forces (5-nm distance) [27]. The possibility of deposition of bacterial cells to the mineral surface can be considered in terms of thermodynamics. However, the thermodynamic approach ignores electrostatic interactions, which does not fully explain the cell adhesion process.

A separate problem, which is not considered in the DLVO theory and thermodynamic approach, is the mineral surface’s heterogeneity. The anisotropic chemical heterogeneity of the mineral surface might play an essential role in cell adhesion. Differences between experimental colloid particle deposition and theoretical values predicted by the DLVO theory might result from the roughness of the solid surface [28]. This phenomenon mostly occurs during the adhesion of *Bacillus subtilis* cells to the kaolinite [29].

As was shown, the adhesion of acidophiles onto the mineral surface can be successfully described by extended DLVO theory. Adsorption of surfactants and biosurfactants changes the surface free energy of the schwertmannite (Table 3). It was observed studying the adhesion of *Escherichia coli* that the number of cells attached to the surface decreased with the decreasing surface free energy of the substrate [13]. Schwertmannite’s surface after the adsorption of rhamnolipid showed the lowest value of surface free energy (31.7 mJ/m^2^), indicating feasible conditions for cell adhesion.

Hydrophobicity is often used in the interpretation of bacterial adhesion. The hydrophobic nature of microorganisms in mining environments varies. This property is significant in selecting bacterial strains in bioleaching processes and others in which cell adhesion to the surface plays a significant role. In combination with the hydrophobicity of solid surfaces, it is considered a dominant characteristic when considered simultaneously with electrokinetic potential.

In general, the $\Delta G_{\mathrm{BSL}}^{\mathrm{TOT}}$ decreases with increasing hydrophobicity. Therefore, the highest adhesion strength was observed in the case of mineral-rhamnolipid interaction, where there was a high hydrophobicity and the lowest Gibbs free energy value. Experimental results show that based only on the thermodynamic approach, adhesion was energetically favorable in all cases. At the same time, the results of the measurement of the potential of the zeta suggested electrostatic repulsion in three of them.

As it is in this study, when the solid and bacterial surface is hydrophilic, a distinct influence of electrostatic interactions is observed [30]. Additionally, with a small ionic strength (10^−3^ M NaCl), the electrostatic force operated at a longer range than the acid-base interaction. This resulted in a high energy barrier to adhesion. Consequently, bacterial adhesion was primarily mediated by the electrostatic force rather than the acid-base interaction, resulting in a negligible hydrophobicity effect on adhesion relative to zeta potential. Therefore, when ionic strength is small, there is a need to consider electrostatic interactions, not only hydrophobicity. Similar remarks were also made by Hong et al. [31]. On this basis, it can be concluded that an extended DLVO theory is the best tool to fully explain initial bacterial adhesion.

Another important aspect of this study is predicting the bacteria’s behavior in contact with the solid surface in an acidic environment. It has been proven that the expansion of acidophiles is facilitated by the presence of schwertmannite on the sulfide mineral surface [32]. Physicochemical conditions of acidic waters favor the stability of schwertmannite. The increase in the thickness of the latter layer facilitates the multiplication of acidophiles, which oxidize iron. The main effect is an increase in the oxidation rate due to an increase in Fe(III) solubility on sulfide–mineral surfaces. In such a situation, the ability to control the adhesion of acidophilic bacterial cells to the surface gives the possibility of counteracting the adverse effects of acid mine drainage. According to the obtained results, the addition of a cationic surfactant (CTAB) at a concentration of 10^−3^ M will prevent the adhesion of acidophiles to the schwertmannite surface in pH 3, thereby inhibiting bioleaching. Contrary, the presence of rhamnolipid (10^−3^ M) will favor the adhesion process and facilitate bioleaching.

There is still a need for studies on surfactant influence on both bacterial cells and minerals, giving a comprehensive view of the phenomenon of the initial step of adhesion in the presence of surfactants. Such information might be important in selecting microorganisms for processes where cellular adherence to a solid surface is crucial.

## 5. Conclusions

We investigated the acidophilic bacteria attachment to the unmodified and modified (by surface-active reagents) schwertmannite surface. The adsorption of CTAB, SDS, and rhamnolipid caused changes in surface properties, which was monitored by the zeta potential and contact angle measurements. Additionally, the surface free energy of both pure and modified schwertmannite surfaces was calculated. The schwertmannite surface had the smallest surface free energy (39.7 mJ/m^2^) after rhamnolipid adsorption. The calculation of adhesion energy using thermodynamic theory showed negative values for all tested suspensions.

Nevertheless, the mineral modified by rhamnolipid conditions was best suited to microorganism adhesion. The negative value of total adhesion energy was −87.64 mJ/m^2^. The total interaction energy, calculated according to the extended DLVO theory, gave a negative value only for schwertmannite-SDS cell and schwertmannite-rhamnolipid cell suspensions. This was in line with detailed experimental results and indicated that the mineral-cell attachment is energetically favored in these two cases. These results have also confirmed that physico-chemical forces, including electrostatic interactions, must be considered to describe adhesion more accurately. Application of extended DLVO theory predicted the presence of energy barriers that bacteria need to overcome to adhere to the mineral. The information obtained is essential in understanding the fate of bacteria in soil and guiding such processes as bioleaching or in-situ bioremediation.

## Figures and Tables

**Table 1:** The zeta potentials of the bacterial cell (ionic strength 10^−3^ M NaCl).

pH	Zeta Potential [mV]	SD *
1.91	+4.8	1.1
2.33	+4.6	1.1
2.57	+4.4	1.4
3.01	+5.0	1.2

* SD—standard deviation.

**Table 2:** The size distribution of schwertmannite particles in the presence of surfactants and biosurfactant.

Size Distribution Parameter	The Concentration of Surface-Active Compound (mM)				
	0.1	0.3	0.5	0.7	1.0
**Schwertmannite-Rhamnolipid**					
Mean (μm)	57.38	48.71	65.93	30.80	110.9
Median (μm)	41.16	29.20	51.17	22.41	58.49
Mode (μm)	37.97	28.70	80.08	31.51	80.07
Span	2.14	2.47	2.05	3.26	8.42
**Schwertmannite-SDS**					
Mean (μm)	24.61	25.70	25.19	25.32	25.87
Median (μm)	21.40	22.34	21.77	21.70	22.40
Mode (μm)	26.15	28.70	28.70	28.70	28.70
Span	1.82	1.84	1.78	1.905	1.916
**Schwertmannite-CTAB**					
Mean (μm)	25.25	27.46	26.32	29.86	28.93
Median (μm)	21.98	21.89	22.03	23.35	22.93
Mode (μm)	26.15	26.15	26.15	28.70	26.15
Span	1.80	1.95	1.86	2.04	2.03

**Table 3:** Contact angle and surface free energy of bacterial cell and pure and modified schwertmannite.

Sample	Contact Angle (^o^)			Surface Free Energy (mJ/m^2^)				
	Water	Diiodo-Methane	Form-Amide	γ^−^	γ^+^	γ^AB^	γ^LW^	γ^TOT^ *
**Bacteria**	47.7	27.3	24.8	23.4	0.937	9.36	45.4	54.8
**Pure Mineral**	17.0	26.0	8.00	49.7	0.600	11.2	45.8	57.0
**Mineral-CTAB**	21.0	35.0	16.0	48.8	0.900	13.4	42.0	55.4
**Mineral-SDS**	17.0	28.0	17.0	52.6	0.40	9.40	45.0	54.4
**Mineral-Rhamnolipid**	100	42.0	61.0	0.40	0.70	1.10	38.6	39.7

* γ^TOT^ = γ^AB^ + γ^LW^.

**Table 4:** The free energy of adhesion (mJ/m^2^) calculated using thermodynamic theory.

Sample	γ_BS_	γ_BL_	γ_SL_	$\Delta G_{\mathrm{BSL}}^{\mathrm{LW}}$	$\Delta G_{\mathrm{BSL}}^{\mathrm{AB}}$	$\Delta G_{\mathrm{BSL}}^{\mathrm{TOT}}$
**Pure Mineral**	−0.8570	6.061	−12.70	−30.99	14.47	−16.52
**Mineral-CTAB**	−0.0165	6.061	−12.60	−28.42	14.02	−14.40
**Mineral-SDS**	−1.623	6.061	−15.30	−33.28	16.06	−17.22
**Mineral-Rhamnolipid**	1.380	6.061	39.60	−49.75	−37.89	−87.64
